# Construction and analysis of competing endogenous RNA ceRNA networks in the liver of black rockfish (*Sebastes schlegelii*) following *Aeromonas salmonicida* infection

**DOI:** 10.1016/j.cirep.2023.200124

**Published:** 2023-12-18

**Authors:** Xiantong Liu, Ningning Wang, Haohui Yu, Xiaoyan Zhang, Min Cao, Chao Li

**Affiliations:** School of Marine Science and Engineering, Qingdao Agricultural University, Qingdao 266109, China

**Keywords:** *Sebastes schlegelii*, *Aeromonas salmonicida*, Liver, circRNA-miRNA-mRNA networks, Immune response

## Abstract

Given the dual roles in immune function and metabolism, the liver emerge as a compelling candidate to bridge host defense and metabolic adjustments during pathogen infections in teleosts. In order to dissect the roles of the liver in the immune response of *Sebastes schlegelii*, we conducted an investigation into the activities of SOD, CAT and GPX4, performed a systematic analysis of circRNA, miRNA and mRNA expression profiles, as well as constructed circRNA-miRNA-mRNA regulatory networks in the liver of *S. schlegelii* following *Aeromonas salmonicida* infection were performed in the present study*.* The present results demonstrated the content of SOD, CAT and GPX4 increased significantly at early infection stage to protect the liver tissue from excessive damage. Additionally, 622 circRNA-miRNAs pairs, 78 miRNA-mRNA pairs and 327 circRNA-miRNA-mRNA pairs were identified in our study. These differently expressed circRNAs and miRNAs were found to be associated with the regulation of *LMNB1, DMBT1, NAMPT, IFIT1, CELSRs, PYGL*. GO and KEGG enrichment analysis revealed that these differently expressed genes were involved in TLR signal pathway, RLRs signal pathway, PPAR signal pathway, shedding light on antibacterial ceRNA networks within the liver of *S. schlegelii* post *A. hydrophila* post infection, which provides new insights into the immune mechanisms of teleost.

## Highlights

• The content of SOD, CAT and GPX4 increased significantly at early infection stage in the liver of *Sebastes schlegelii*.
• Totally, 622 circRNA-miRNAs pairs, 78 miRNA-mRNA pairs and 327 circRNA-miRNA-mRNA pairs were identified in *Sebastes schlegelii*.
• These differently expressed circRNAs, miRNAs and mRNAs were related with the regulation of *LMNB1, DMBT1, NAMPT, IFIT1, CELSRs, PYGL*.
• GO and KEGG enrichment analysis showed that differently expressed genes are related with immunity signal pathways.
• To validate the accuracy of RNA sequencing, we detected the expression levels of 30 DE-RNAs by qRT-PCR.

## Introduction

The liver is considered to be the primary organ that involved in metabolism, situated within abdominal cavity between the intestine and systemic circulation, thereby constantly exposed to nutritional damage, intestinal microbiome products, and toxic substances [1]. After absorption by the intestine and transportation to the liver, nutrients are filtered for excess harmful substances, making the liver an immune modulator in addition to its metabolic functions T [2]. It has been demonstrated that the vertebrate liver has been shown to produce cytokines, chemokines, complement components and acute phase reactants proteins in response to pathogen infection [3]. Given the dual roles in immune function and metabolism, liver can be selected as an interesting candidate to bridge host defense and metabolic adjustments during pathogen infection in teleosts. For instance, Castro et al. confirmed the presence of *IgM^+^, IgD^+^, IgT^+^, CD8^α+^, CD3^+^* cells, as well as cells expressing the major histocompatibility complex (MHC-II) in the liver of rainbow trout (*Oncorhynchus mykiss*), and further evaluated the immune role of liver tissue in response to viral attack [4]. Similarly, transcriptome analysis conducted in *Epinephelus akaara* revealved immune-related genes such as *IL8, TLR9, CXCR4, CCL4,* and *IκBα* were found in the liver, suggesting a high carbohydrate level of diet can lead to inflammatory immune response in the liver of *E. akaara* [5]. Therefore, identification of candidate genes involved in the liver immunity and metabolism is crucial for elucidating its molecular mechanism.

High-throughput sequencing enables comprehensive molecular profiling of global gene expression views, encompassing mRNA and non-coding RNAs such as long ncRNAs (lncRNAs), circular RNAs (circRNAs) and microRNAs (miRNAs). Among these ncRNAs, circRNA has been shown to participate in various biological processes via biding to miRNA as a sponge, thereby influencing the expressions of the downstream target genes of miRNA and relevant signaling pathways [6], [7], [8]. Recent advances in high-throughput sequencing technology have lead to the identification of numerous potential regulatory pathways including lncRNA/circRNA-miRNA-mRNA interactions in teleost. For instance, 1947 differentially expressed mRNAs, 9 differentially expressed miRNAs, and 4 differentially expressed circRNAs between fast- and slow-growing individuals of Nile tilapia (*Oreochromis niloticus*) were observed. Based on the constructed ceRNAs networls, it was found that circMef2c can interact with 3 miRNAs and 65 mRNAs, thus providing novel insights into the role of circRNAs in the regulation of muscle growth in teleost (Golam [9]). In teleost, infection-associated ceRNAs have been reported in a number of species. Cai et al. investigated the whole-transcriptome of the *Vibrio anguillarum* infected turbot liver, and constructed miRNA-circRNA pairs, miRNA-mRNA pairs and 65 circRNA-miRNA-mRNA pairs. They speculated that novel_circ_0002878/miR-34a/NR1D2 axis may be involved in protection against bacterial infections (**Cai et al.** [10]). Similarly, researchers investigated the expression profiles of circRNAs, miRNA and mRNA in *S. schlegelii* response to *Edwardsiella tarda* infection, and identified ceRNAs networks that are strongly related to immune signaling pathways, such as NF-κB signal pathway and chemokine signaling pathway [6]. In addition, whole-transcriptome sequencing was conducted in the spleen of black rockfish following *Aeromonas salmonicida* challenging to construct circRNA-miRNA-mRNA networks. Totally, 290 circRNA-miRNA-mRNA networks were constructed including 31 circRNAs, 50 miRNAs, and 156 mRNAs, which can regulate immune related genes and signal pathways such as immunoglobulin, mucin domain-containing protein 4, Galectin-9 and Cathepsin D, FoxO signaling pathway, Jak-STAT signaling pathway, TGF-β signaling pathway [11]. However, an investigation on the interactions of mRNAs and ncRNAs in the liver of *S. schlegelii* response to *A. salmonicida* have not been carried out systematically.

Black rockfish (*S. schlegelii*), which inhabits the coastal waters of China, Japan, and Korea, has been extensively studied due to its viviparous breeding habits and aquaculture requirements. However, the aquaculture of *S. schlegelii* stillfaces several shortcomings such as low level of farming intensification, inadequate breeding techniques and degradation of germplasm resources, which have directly led to decrease in its growth rate, disease resistance and mortality. The liver plays an important role in fish metabolism, immune defense and life activities. Therefore, *S. schlegelii* was selected as the object to explore the changes in the activities of glutathione peroxidase 4 (*GPX4*), superoxide dismutase (*SOD*) and catalase (*CTA*) in liver and the whole transcriptome expression characteristics following *Aeromonas salmonicida* infection at different time points. The objective of this study is to investigate the immune response of *S. schlegelii* to bacterial infections based on the gene expressions as well as enzyme activities.

## Materials and methods

### Ethics approval and consent to participate

The protocol was approved by the Committee on the Ethics of Animal Experiments of Qingdao Agricultural University IACUC (Institutional Animal Care and Use Committee).

### Sample collection and bacterial infection

The experimental healthy fish were obtained from a local fish farm in Yantai, Shandong Province. Prior to the bacterial infection experiment, fish were acclimated in a recirculating fresh water system for one week before bacterial infection experiment was conducted. For bacterial infection, the experimental groups of fish were challenged in triplicate 30 L (20 L water) aquaria, with each replicate consisting of 5 randomly selected individuals. The individuals were immersed in *A. salmonicida* for 2 h at a final concentration of 5–6 × 10^7^ CFU/mL. Subsequently, liver tissues from 5 fish were collected as one sample at 2 h (AS2H), 12 h (AS12H) and 24 h (AS24H) after euthanized with MS-222 (200 mg/L), respectively. Meanwhile, fish in seawater were defined as the control (CON) group. All samples were flash frozen in liquid nitrogen and stored at − 80 °C for RNA extraction.

### Measuring of the activities of SOD, CAT and GPX4 enzyme

After collecting tissues from different time points, 0.1 of liver sample of the *A. salmonicida* infected *S. schlegelii* was homogenized in 2 mL of 50 mM (containing 0.2 mM EDTA) phosphate-buffered saline (PBS; pH 7.8). The homogenate was then transferred to a test tube and centrifuged at 12, 000 rpm for 20 min. After centrifugation, the supernatant was collected as the enzymatic solution for the following measurement of the activities of SOD, CAT and GPX4 enzyme. The activities of SOD, CAT and GPX4 enzyme were detected by using a SOD kit (Jiancheng, A007-2-1), CAT (Jiancheng, A001-1) and GPX4 kit (Jiancheng, H545-1-1), respectively.

### Library construction and sequencing

Total RNA was extracted from control and infected liver samples using TRIzol Reagent (Invitrogen, Carlsbad, CA, USA) according to the manufacturer's instructions. The purity, and integrity of extracted RNA were assessed using NanoPhotometer spectrophotometer (IMPLEN, CA, USA) and Agilent 2200 TapeStation (Agilent Technologies, USA), respectively. Subsequently, the library construction, sequencing, gene expression levels quantification, ceRNA networks constructions, and functional analysis have been described in a previous study [6].

### qRT-PCR verification of the expressions of circRNA, miRNA and mRNA

To validate the accuracy of RNA sequencing, we assessed the expression levels of 30 DE-RNAs, including 10 DE-circRNAs, 10 DE-miRNAs, and 10 DE-mRNAs using qRT-PCR, as well as compared the expression patterns with those from RNA sequencing results. The same RNA samples used for library construction were utilized in this analysis. PrimerQuest (https://sg.idtdna.com/PrimerQuest/Home) was used to design primers for all DE-mRNAs and DEcircRNAs. For miRNA primers, they were designed on the basis of the instructions of miRcute miRNA isolation kit (Tiangen Biotech, China). Subsequently, β-actin was chosen as an internal control to normalize the relative quantification of circRNAs, and mRNAs, while U6 was used as an internal control of miRNA. The used primers in this study were listed in Table 1. The amplifications of all primers were ranged from 95 % to 105 %. Expression profiles of the selected-genes were analyzed with a CFX96 real-time PCR detection system (Bio-Rad Laboratories, Hercules, CA, USA) as descripted in previous study (**Cao et al.** [12]). Finally, the 2^−∆∆Ct^ method [13] was used to calculate the relative expression levels and data were expressed as the mean ± standard error of three replicates.

**Table 1:** Primers used in the current study.

Primer	Sequence (5′−3′)
dre-miR-155	TTAATGCTAATCGTGATAGGGG
dre-miR-190a	TGATATGTTTGATATATTAGGT
dre-miR-22a-3p	AAGCTGCCAGCTGAAGAACTGT
dre-let-7b	TGAGGTAGTAGGTTGTGTGGTT
dre-miR-301b-5p	GCTTTGACGATGTTGCACTAC
dre-miR-128–3p	TCACAGTGAACCGGTCTCTTTT
novel_169	TCAGGAGTTTTAGAAATCGGTG
novel_559	TAGGACAATAATTAAGGCAGA
dre-miR-456	CAGGCTGGTTAGATGGTTGTCA
dre-miR-20a-5p	TAAAGTGCTTATAGTGCAGGTAG
TCONS_00098868F	TGTACGATGGATGCCGTAAAG
TCONS_00098868R	GGATCAGCTTTGAACAGGAAATG
TCONS_00019905F	CAAACATGGTGGCCCTTTAATC
TCONS_00019905R	CAGCCATCTTCAGGGTCATATC
TCONS_00070177F	AGTCTGGTGGTTCTGCTTTC
TCONS_00070177R	TCTGCTGTGACCTTCTGTAATG
TCONS_00113123F	CCCGTGTCTTTCTACCTCTTTC
TCONS_00113123R	CGACTTCGGCAGAGTCAAAT
TCONS_00088456F	CTGAGGAGGAGATGTGAATGTG
TCONS_00088456R	ACTCTGTCCCTCTGTGATGTA
TCONS_00009109F	GACTGCTACAACCACGACTAC
TCONS_00009109R	GCTCTCAGTCCACAACCATT
TCONS_00044066F	GTGAGGGCTGTTTGTTAGGA
TCONS_00044066R	CGCCCTCACCACCATTATTA
TCONS_00002961F	GTCGTGGTTACCAGGTGTATAAG
TCONS_00002961R	CGATGGTCTTAACCTCGTTCTC
evm.model.Chr11.381F	ACAATGTCGGGTTCCTCTAATC
evm.model.Chr11.381R	GGGAGACCTTCACTCTGTTTATG
TCONS_00090561F	GTTGCTCAGCTGGTCTGATAG
TCONS_00090561R	TGACTGACTGCTGGATGATTG
novel_circ_0000790F	ACCTCTCGGCTGTCTGTAT
novel_circ_0000790R	CAGCTCATTGAAGCGGATTTG
novel_circ_0000829F	TTACCTCTACCCAGATCCATCC
novel_circ_0000829R	AGTGCAGTTAGACACCCAATAC
novel_circ_0000791F	GCACAGAGAAGAAGAGTGTGAG
novel_circ_0000791R	GAAGAGGGAGGAGGAGAGAAG
novel_circ_0000320F	TTGGGTCTGAAGGTCAAAGAG
novel_circ_0000320R	GGTGGAGTAGGACAGGTAGAA
novel_circ_0000773F	TGTGGGATTCCTCCTCTCAA
novel_circ_0000773R	GGGACACTCTCTCTCCAATCT
novel_circ_0001459F	CACCAACAACACCGAATGTG
novel_circ_0001459R	CTCCCTGTGTGGCATCTTATC
novel_circ_0000343F	TTGGAGGACGTGAAGAGTTTG
novel_circ_0000343R	ACAAGTGATGTAGGACGGATTG
novel_circ_0001460F	CCAGTCTGTGCTTTCCAGAT
novel_circ_0001460R	AGCAGTTGTGTTTGTGTTTCC
novel_circ_0000004F	GGATGGAGAGCACGACAAA
novel_circ_0000004R	CAGTTCTTCCTCCGGATCTTATC
novel_circ_0000298F	GGAACACGCCGATAGAAGAG
novel_circ_0000298R	CCAGCAGGTGGGATGTATTT

### Data analysis

The physiological and biochemical indexes and fluorescence quantitative experiments were repeated for 3 times. SPSS 20.0 was used for statistical significance analysis, and the data were expressed as mean ± standard deviation. *p* < 0.01 is considered to be a significant difference and *p* < 0.05 was considered a significant difference.

## Results

### Activities of SOD, CAT and GPX4 enzyme

**Fig. 1:**
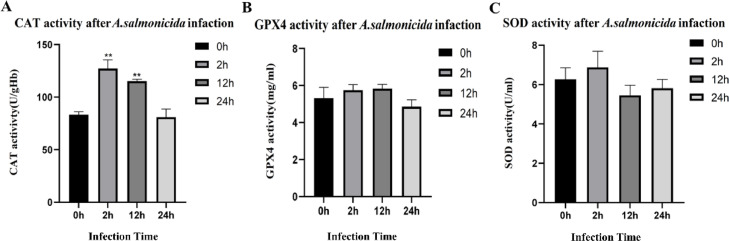
The CAT, GPX4 and SOD activities in the liver of *S. schlegelii* following *A. salmonicida* infection.

The activities of SOD, CAT and GPX4 enzyme in control and *A. salmonicida* infected livers of *S. schlegelii* was shown in Fig. 1. The initial activity of CAT in the liver of healthy fish (0 h) was measured as 83.32 ± 2.65 U/gHb. Upon infection with *A. salmonicida,* the content of CAT in *S. schlegelii* reached its peak level at 127.20 ± 8.25 U/gHb after 2 h infection, followed by a decreased in subsequent infected groups over times. In detail, the activities of CAT were recorded as 115.21 ± 1.88 U/gHb at 12 h and reached the lowest level (80.95 ± 7.70 U/gHb) at 24 h post-infection (Fig. 1A). Additionally, we also observed that the activity of GPX4 was 5.32 ± 0.59 mg/ml at 0 h, which increased to 5.75 ± 0.30 mg/ml at 2 h after infection, and then continued to increase, reaching the highest level at 12 h (5.83 ± 0.23 mg/ml) (Fig. 1B). Similarly, the activity of GPX4 also decreased at 24 h (4.86±0.36 mg/ml). SOD activity was 6.26 ± 0.58 U/ml at 0 h, reached the highest level (6.87 ± 0.82 U/ml) at 2 h after infection. Nevertheless, the decline observed for SOD levels during subsequent time points i.e., 12 h and 24 h, was less pronounced compared to that seen within the control group (Fig. 1C).

### Transcriptome sequencing results of circRNAs

**Fig. 2:**
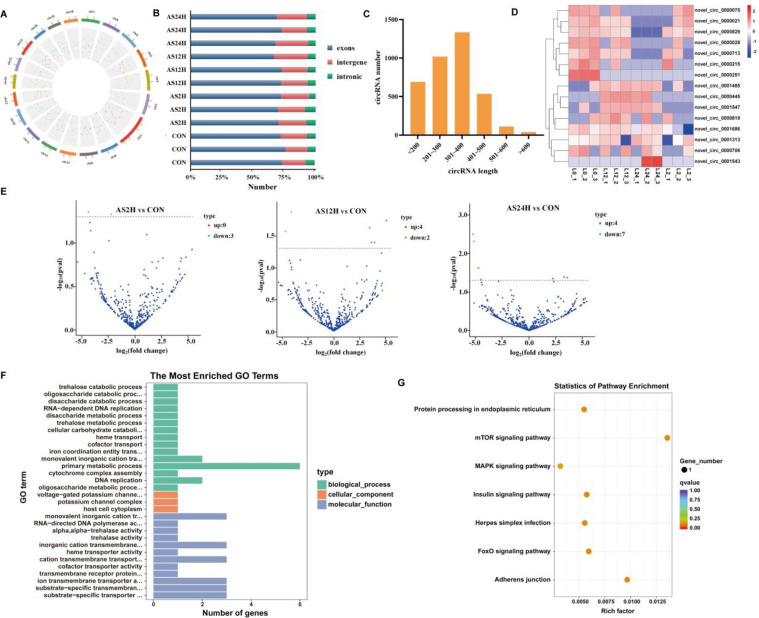
Features and characteristics of circRNAs in the liver of *S. schlegelii* following *A. salmonicida* infection. (A, B) Chromosome locations of circRNAs; (C) The length distributions of identified circRNAs; (D) Expression patterns of circRNAs among control and infected groups; (E) Volcano plots of DE circRNAs among control and infected groups; (F) GO term analysis of DE circRNAs; (G) KEGG analysis of DE circRNAs.

In order to gain a comprehensive understandingof the circRNAs in response to *A. salmonicida* infection of *S. schlegelii*, the rRNA-depleted samples from control and *A. salmonicida* infected liver samples at different infection time points (2 h, 12 h and 24 h) were used to library construction and sequencing. A total of 2406 circRNAs were identified in *S. schlegelii* that were widely distributed on different chromosomes (Fig. 2A). Among these circRNAs, 71.60 %, 0.22 % and 0.07 % identified circRNAs were originated from exons, intergenic and intron, respectively (Fig. 2B). The length distribution of circRNAs were mainly ranged from 200 to 400 bp (Fig. 2C). As shown in Fig. 2D, the expression patterns of the *A. salmonicida*-infected and control samples were categorized into different clusters. Furthermore, the results of circRNAs expression patterns showed that a total of 3, 6 and 11 DE-circRNAs were identified in *A. salmonicida*-infected groups (AS2H, AS12H, and AS24H) against the control group (Fig. 2E). Functional analysis was performed to clarify the biological function of circRNAs of *S. schlegelii* after *A. salmonicida* infection (Fig. 2F, G). The results showed that the DE-circRNAs were involved in multiple biological processes such as protein processing in edoplasmic reticlum, mTOR signaling pathway, MAPK signaling pathway, insulin signaling pathway, herpes simplex infection, FoxO signaling pathway, adherens junction.

### Transcriptome sequencing results of miRNAs

**Fig. 3:**
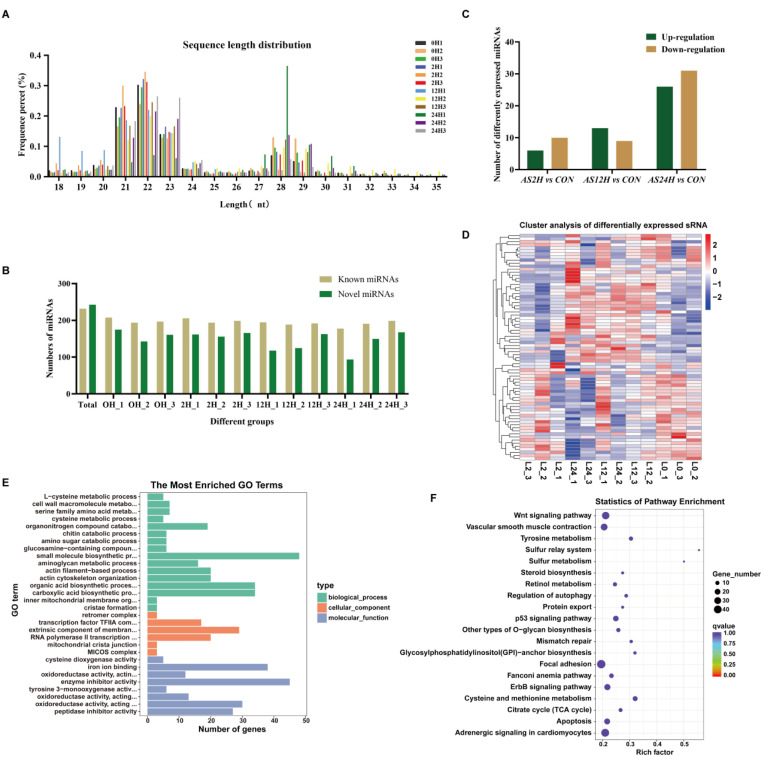
Features and characteristics of miRNAs in the liver of *S. schlegelii* following *A. salmonicida* infection. (A, B) The length distribution of identified miRNAs; (C) The differently expressed miRNAs; (D) Expression patterns of miRNAs among control and infected groups; (E) Go term analysis of DE-miRNAs; (F) KEGG analysis of DE-miRNAs.

The length of these miRNAs was mainly ranged from 21 to 23 nt, with a peak distribution observed at 22 nt (Fig. 3A). A total of 473 miRNAs were obtained, including 231 known miRNAs and 242 novel miRNAs (Fig. 3C). Among which, 16 (AS2H), 22 (AS12H) and 57 (AS24H) differentially expressed miRNAs were identified, respectively (Fig. 3B). The top DE-miRNAs were presented in a heat map based on gene expression (Fig. 3D).To further explore the functions of these DE-miRNAs furtherly, GO and KEGG were used to perform statistical analysis of their target genes. The target genes were primarily enriched in 2436 GO term processes (Fig. 3E). Additionally, KEGG analysis showed that the target genes of DE-miRNAs were involved in Wnt signaling pathway, focal adhesion and adrenergic signaling in caridiomyocytes (Fig. 3F).

### Statistical analysis of mRNAs data

**Fig. 4:**
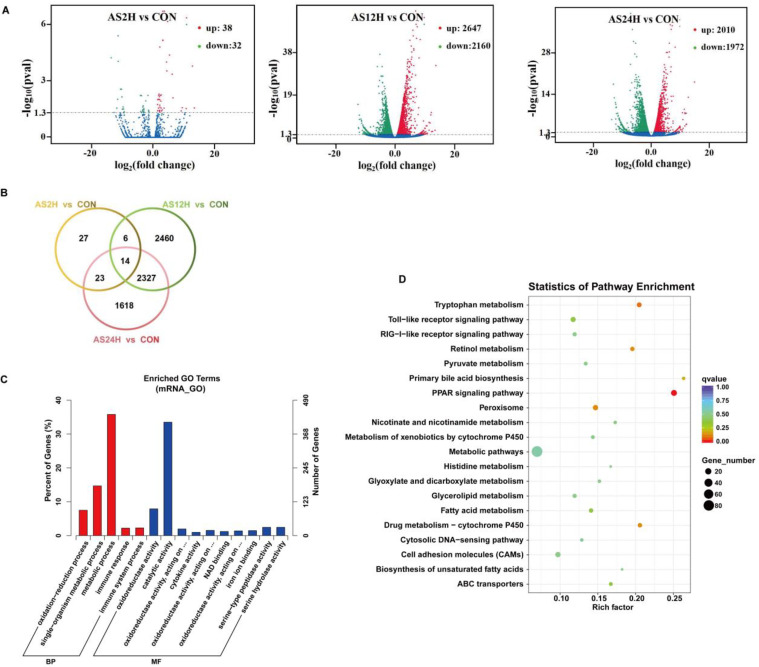
Features and characteristics of mRNAs in the liver of *S. schlegelii* following *A. salmonicida* infection. (A) Volcano plots were drawn to visualize the standardized expression of mRNAs between the infected and control groups. The red and green points represent differentially expressed mRNAs with statistical significance (*P* < 0.05); (B) Veen diagram of mRNAs; (C) Go term analysis of DE-mRNAs; (D) KEGG analysis of DE-mRNAs.

A total of 8859 significantly DEmRNAs were identified by stringent thresholds (FDR< 0.05). Among which, 4695 DEmRNAs were upregulated, and 4164 DEmRNAs were downregulated (Fig. 4A). We also found that 14 core genes were expressed differently at each time point of infection, and 27, 2460 and 1618 genes that specific to AS2H, AS12H and AS24H, respectively (Fig. 4B). Then, the function of DE-mRNA was analyzed by GO analysis and KEGG pathway analysis showed that most DE-mRNAs participate metabolic process. In addition, we also noticed some genes are related with TLR signal pathway, RLRs signal pathway, PPAR signal pathway (Fig. 4C, D).

### Construction of the circRNA-miRNA, miRNA-mRNA, and ceRNA regulatory networks

**Fig. 5:**
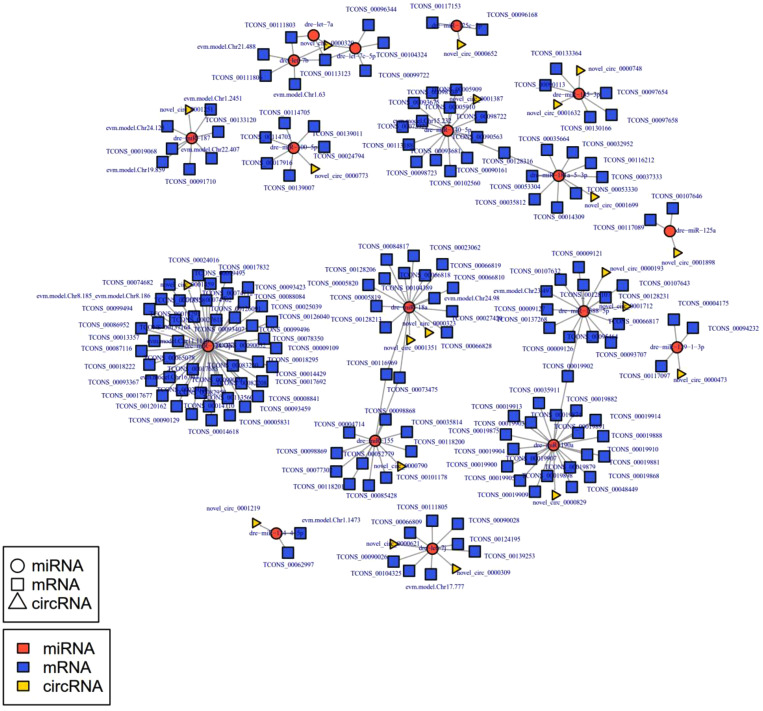
The ceRNA regulatory networks in the liver of *S. schlegelii* following *A. salmonicida* infection. Red circle nodes represent miRNAs, yellow triangle nodes represent circRNAs, and blue squares represent mRNAs.

Among ncRNAs, circRNAs can regulate the gene expression on post-transcriptional levels by sponging miRNAs. Simultaneously, miRNAs can bind to the 3′ UTR of mRNAs or the coding regions to repress or degrade the mRNAs, thus influencing the expression of relative genes. Therefore, the circRNA-miRNA, miRNA-mRNA, and ceRNA regulatory networks during the *A. salmonicida* infection process in the liver of *S. schlegelii* were analyzed. Totally, 622 circRNA-miRNAs pairs were identified in our study, including 164 circRNAs and 175 miRNAs (Table S1). And, 78 miRNA-mRNA pairs were identified, including 43 miRNA and 69 mRNA (Table S2). The ceRNA regulatory network encompassed 327 circRNA-miRNA-mRNA pairs, including 31 circRNAs, 35 miRNAs, and 31 mRNAs (Fig. 5 and Supplementary Table S3). Among which, we identified several circRNA-miRNA-mRNA networks that could be considered as potential candidates for future functional analysis, including novel_circ_0000320/dre-let-7b/evm.model.Chr1.63 (Lamin-B1, LMNB1), novel_circ_0000320/dre-let-7a/TCONS_00113123 (GTPase IMAP family member 8-like, GIMAP8), novel_circ_0001251/dre-miR-187/evm.model.Chr1.2451 (FERM domain-containing protein 4B, FRM4B), novel_circ_0000773/dre-miR-100–5p/TCONS_00017916 (collagenase 3-like), dre-miR-145–3p/novel_circ_0000748/TCONS_00097654 (deleted in malignant brain tumors 1 protein-like, DMBT1), novel_circ_0001351/dre-miR-18a/TCONS_00116969 (dixin-A-like), novel_circ_0000791/dre-miR-22a-3p/evm.model.Chr1.1161 (Nicotinamide phosphoribosyltransferase, NAMPT), novel_circ_0001377/novel_215/evm.model.Chr1.1906 (Cadherin EGF LAG seven-pass G-type receptor 2, CELR2), novel_circ_0000790/dre-miR-155/TCONS_00116969 (3-oxoacyl-[acyl-carrier-protein] reductase, FabG-like), novel_circ_0001459/dre-miR-128–3p/TCONS_00078350(ubiquitin carboxyl-terminal hydrolase 47-like, USP47), novel_circ_0001387dre-miR-140–5p/TCONS_00102560 (interferon-induced protein with tetratricopeptide repeats 1-like, IFIT1), novel_circ_0000004/dre-miR-456/evm.model.Chr11.381 (Guanine nucleotide-binding protein, GBG5), novel_circ_0001880/novel_182/evm.model.Chr15.1085 (Glycogen phosphorylase, PYGL), novel_circ_0000791/dre-miR-22a-3p/evm.model.Chr16.719 (Pumilio homolog 1, PUM1), novel_circ_0000342/dre-miR-22b-3p/evm.model.Chr3.156 (Myotubularin-related protein 7, MTMR7), novel_circ_0001377/novel_215/evm.model.Chr24.291 (Neural-cadherin, CADN) etc. These circRNA-miRNA-mRNA networks can be considered as potential candidates for future functional analysis.

### Validation of circRNAs, miRNAs and mRNAs by qRT-PCR

**Fig. 6:**
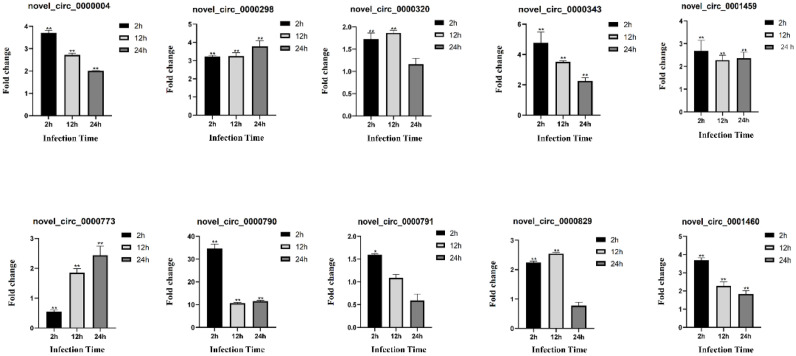
Validation of circRNAs by qRT-PCR. Error bars represent SE of expression levels of each circRNA. The relative expression level of circRNAs in 2 h, 12 h and 24 h were calculated as the ratio of the gene expression level (qRT-PCR). * on the bars represent *p* < 0.05 and ** represent *p* < 0.01 between *A. salmonicida* infected *S. schlegelii* and control groups (*n* = 3 for each group).

**Fig. 7:**
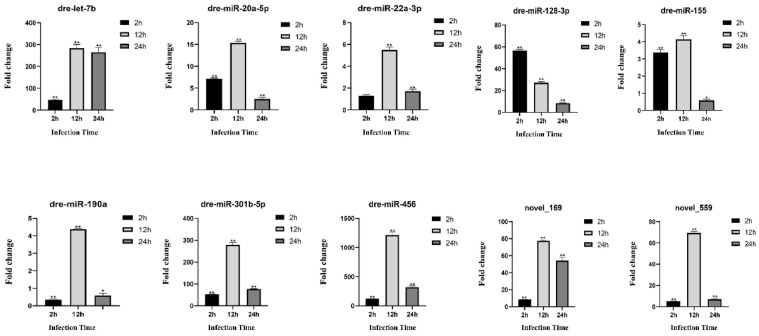
Validation of miRNAs by qRT-PCR. Error bars represent SE of expression levels of each miRNA. The relative expression level of miRNAs in 2 h, 12 h and 24 h were calculated as the ratio of the gene expression level (qRT-PCR). * on the bars represent *p* < 0.05 and ** represent *p* < 0.01 between *A. salmonicida* infected *S. schlegelii* and control groups (*n* = 3 for each group).

**Fig. 8:**
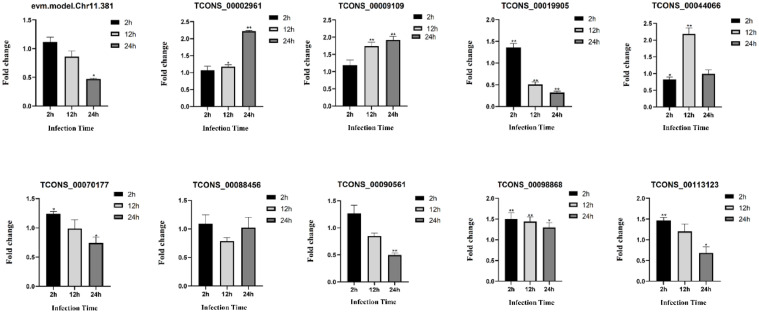
Validation of mRNAs by qRT-PCR. Error bars represent SE of expression levels of each mRNA. The relative expression level of mRNAs in 2 h, 12 h and 24 h were calculated as the ratio of the gene expression level (qRT-PCR). * on the bars represent *p* < 0.05 and ** represent *p* < 0.01 between *A. salmonicida* infected *S. schlegelii* and control groups (*n* = 3 for each group).

In order to authenticate the authenticity of the DE-circRNAs, DE-miRNAs and DE-mRNAs that identified from the transcriptome data of *S. schlegelii* after *A. salmonicida*, 10 differentially expressed circRNAs were selected for validation. The qRT-PCR results confirmed that the expression patterns of the selected circRNAs were consistent with the sequencing results. It is noteworthy that most of the 10 detected circRNAs were up-regulated in *S. schlegelii* after *A. salmonicida*. For instance, the expression of novel_circ_0000298 increased by 3.23 fold, 3.24 fold and 3.79 fold at 2 h, 12 h and 24 h after infection, respectively. novel_circ_0000343 expression was up-regulated 4.78 fold at 2 h after infection, 3.51 fold at 12 h after infection, and 2.26 fold at 24 h after infection. While, the expression of novel_circ_0000773 was decreased by 0.55 fold during early stages of infection but demonstrated an upward trend as infection time increased (Fig. 6). Meanwhile, the similar expression trends between sequencing analysis and qRT-PCR of 10 miRNAs were present though there were few differences in the fold change of expressions. miRNAs changed significantly after infection. For example, the expression of re-let-7b showed an up-regulation trend at all time points, and the up-regulation rate was 46.49 fold at 2 h after infection, 282.76 fold at 12 h after infection, and 265.06 fold at 24 h after infection. The expression of dre-miR-190a was down-regulated by 0.34 fold at 2 h after infection, up-regulated by 4.94 fold at 12 h after infection, and down-regulated by 0.59 fold at 24 h after infection (Fig. 7). The qRT-PCR results confirmed that the expression patterns of the mRNAs were consistent with the sequencing results (Fig. 8). Different mRNAs displayed distinct expression patterns during infection. For example, the expression level of TCONS_00002961 showed an up-regulated trend throughout the entire process, and was up-regulated 1.07 fold, 1.18 fold, and 2.22 fold at 2 h, 12 h, and 24 h after infection, respectively. However, the expression of TCONS_00019905 was up-regulated by 1.36 fold at 2 h after infection, and down-regulated at 12 h and 24 h by 0.51 fold and 0.32 fold, respectively.

## Discussion

*S. schlegelii*, a demersal fish widely distributed in the northwest Pacific along the coast of China, Japan and the Korean Peninsula [14], has been extensively studied with a focus on its growth, behavior, physiology, immunity, and population genetics [[15], [16], [17], [18]]. It has been reported that bacterial and viral pathogens threatened the yield of *S. schlegelii*. Among many pathogenic bacteria, *A. salmonicida* is the causative agent to be linked to fish disease that characterized by high mortality and morbidity [19]. It has been mentioned that the liver is the primary immune tissue of teleost for defensing against pathogentic infections [2]. To gain insights into the immune response mechanisms of liver in this species, the activities of GPX4, SOD and CTA in the liver, as well as whole transcriptome analysis in *S. schlegelii* after challenge with *A. salmonicida* were investigated. These findings provide novel knowledge about ncRNAs in immune responses process in *S. schlegelii*, and will serve as important resources for further investigating the roles of ncRNAs during pathogen infections in teleost.

In this study, we investigated the impact of *A. salmonicida* on the liver of *S. schlegelii*, and detected the activities of SOD, CAT and GPX4. The present results demonstrated the content of SOD, CAT and GPX4 increased significantly at early infection stage. It has been reported the antioxidant enzyme defense system involves SOD, CAT, GPX and glutathione reductase (GSR) [20]. CAT is the hallmark enzyme of peroxisome that widely existed in prokaryotes and eukaryotes, accounting for about 40% of the total peroxisome enzymes, and is one of the key enzymes in the biodefense system established during the process of biological evolution. It acts synergistically with GPX to eliminate hydrogen peroxide generated by superoxide anion through free radical scavenging processes, thereby safeguarding cells from potential damage caused by reactive oxygen species [21]. We thus hypothesized that in the initial stages of infection, the activity of these three enzymes increases rapidly, interacting with reactive oxygen species in *S. schlegelii* to protect the liver from excessive damage.

There is mounting evidence demonstrated that activation and termination of immune responses are regulated at multiple levels, encompassing transcriptional and post-transcriptional levels. At the transcriptome level, a total of 6748 mRNAs were induced differential expression following *A. salmonicida* challenge, with a substantial proportion being classified as immune-related genessuch as apoptosis, C-X-C motif chemokines, cell adhesion molecules, RLRs-I-like receptor, TLR-like receptor, NOD-like receptor. The results demonstrated that these DE mRNAs were induced to participate in a series of biological processes and played immune roles against the invasion of *A. salmonicida*. At the post-transcriptional level, ncRNAs were involved in the interactions between pathogens and teleost [22]. Among which, circRNAs or lncRNAs can competitively bind miRNAs to achieve the purpose of regulating mRNA levels [23]. In our study, we identified 622 circRNA-miRNAs pairs, 78 miRNA-mRNA pairs were identified in our study. Among which, several key immune response pathways regulated by circRNAs and miRNAs were found through functional enrichment analysis. For instance, the JAK/STAT signaling pathway, p53 signaling pathway, Wnt signaling pathway and TLR receptor signaling pathway play crucial roles in the immune system ([24,25]. Furthermore, target genes of DE miRNAs that related to fatty acid degradation, as well as other glycan degradation also been found in the liver of *S. schlegelii*. It has been reported that the degradation of fatty acids is mainly in the liver, mainly because the liver plays a vital role in lipid metabolism [26]. Moreover, we identified several miRNAs that associated with SOD, CAT and GPX4 according to the function of target genes. For instance, the downregulation of novel_11, dre-miR-205–5p and dre-miR-301b-5p may relate to the upregulation of SOD, CAT and GPX4, respectively. Therefore, we hypothesized that these miRNAs can regulate the expression and content of SOD, CAT and GPX4.

Previous studies have demonstrated that the liver harbors populations of immune cells that contributing to immune function including monocytes, macrophages, neutrophils, B lymphocytes, T lymphocytes, NK cells and NKT cells in the body [1]. Based on the transcriptome sequencing technology, numerous of non-coding RNA and mRNA networks were identified in the liver of teleost. For instance, in the infected liver of blunt snout bream (*Megalobrama amblycephala*)*,* the parental genes of 106 differentially expressed circRNAs were enriched in phagocytosis, complement and coagulation cascades, and Fc gamma R-mediated phagocytosis pathways [27]. Furthermore, Cai et al. performed a comprehensive analysis of whole-transcriptome sequencing in the turbot liver following *V. anguillarum* infection, and identified 65 circRNA-miRNA-mRNA networks that related with *TRI25, NR1D2, CMTA1* and *MGLL* [10]. In our study, we totally identified 327 circRNA-miRNA-mRNA regulatory networks including 31 circRNAs, 35 miRNAs, and 31 mRNAs. Among these networks, *LMNB1* was predicted to be regulated by novel_circ_0000320 and dre-let-7b. It has been reported that *LMNB1* is a major structural component of the nucleus that appears to be involved in the regulation of many nuclear functions [28]. The "up-down-up'' relationships of novel_circ_0000320/dre-let-7b/ *LMNB1* suggested that the novel_circ_0000320 can release the inhibition of dre-let-7b and promote the expression of *LMNB1*.We also found that the expression of *DMBT1* was regulated by dre-miR-145–3p, whereas dre-miR-145–3p can be sponged by circRNA novel_circ_0000748. *DMBT1* is a natural defense protein involved in innate immunity, inflammation and epithelial cell differentiation, and plays an important role in diseases associated with pathological processes [29]. We observed that *NAMPT* can be induced and regulated by novel_circ_0000791 and dre-miR-22a-3p in the infected liver. *NAMPT*, also known as pre-B cell clonal enhancer factor and visceral adipose hormone, has become a research hotspot in the fields of nicotinamide adenine dinucleotide biology, metabolism and inflammation due to its various functions in recent years [30]. Moreover, we discovered that dre-miR-140–5p acts as an inhibitor of *IFIT1*, which is regulated by interferon, a variety of viruses and some pathogen-related molecular patterns, which can inhibit viral replication and inhibit inflammatory response [31]. Our findings demonstrate that CELSRs participate in liver cell differentiation and contraction under the regulation of circRNAs and miRNAs. These processes are crucial for multiple biological events during embryonic development, including neuronal/endocrine cell differentiation, vascular valve formation, cell adhesion, and control of planar cell polarity [32]. Additionally, the "down-up-down'' expression patterns of novel_circ_0001880/novel_182/PYGL indicated that gene related to glycogen metabolism was inhibited, and circRNAs and miRNAs participate in this regulatory process. GO and KEGG enrichment analyses showed that differently expressed genes are related with TLR signal pathway, RLRs signal pathway, PPAR signal pathway etc. The present findings unveil antibacterial competing endogenous RNAs network in the liver of *S. schlegelii* following *A. hydrophila* infection, thereby offering novel insights and evidence into the immune mechanisms of teleosts..

## Conclusions

In conclusion, we investigated the circRNA, miRNA and mRNA expression profiles of the *S. schlegelii* liver that challenged with *A. hydrophila* in this study, thereby expanding our understanding of ceRNAs and their roles in teleost. Furthermore, we predicted immune genes that were regulated at the transcriptional and post-transcriptional levels in the liver infected with by pathogenic bacteria based on the constructed ceRNA networks. Meanwhile, the activities of antioxidant enzymes, fatty acid metabolism and glycogen metabolism in the liver were also affected. These findings provided new information for the study of the regulatory mechanisms of immune response of liver in teleost following bacterial infections.

## Funding

This work was supported by the Young Experts of Taishan Scholars (NO.tsqn201909130), and Shandong Technical System of Fish Industry (SDAIT-12-03).

## Availability of data

Not applicable.

## CRediT authorship contribution statement

**Xiantong Liu:** Data curation, Formal analysis, Writing – original draft, Investigation, Methodology. **Ningning Wang:** Formal analysis, Writing – original draft. **Haohui Yu:** Software, Investigation, Methodology. **Xiaoyan Zhang:** Resources, Software, Formal analysis. **Min Cao:** Validation, Formal analysis, Writing – review & editing. **Chao Li:** Conceptualization, Visualization, Writing – review & editing.

## Declaration of Competing Interest

The authors declare that they have no known competing financial interests or personal relationships that could have appeared to influence the work reported in this paper.

## Data Availability

Data will be made available on request.

## Appendix Supplementary materials

Image, application 1

Image, application 2

Image, application 3
